# LGI1 expression and human brain asymmetry: insights from patients with LGI1-antibody encephalitis

**DOI:** 10.1186/s12974-018-1314-2

**Published:** 2018-09-25

**Authors:** Yoonhyuk Jang, Soon-Tae Lee, Ji-Yeon Bae, Tae-Joon Kim, Jin-Sun Jun, Jangsup Moon, Keun-Hwa Jung, Kyung-Il Park, Sarosh R. Irani, Kon Chu, Sang Kun Lee

**Affiliations:** 1 Department of Neurology, Seoul National University Hospital, 101, Daehak-ro, Jongno-gu, Seoul, 03080 South Korea; 2 Department of Neurology, National Center for Mental Health, Seoul, South Korea; 3 Department of Neurology, School of Medicine, Kyungpook National University, Kyungpook National University Chilgok Hospital, Daegu, South Korea; 4 Department of Neurology, Seoul National University Healthcare System Gangnam Center, Seoul, South Korea; 5 Oxford Autoimmune Neurology Group, Nuffield Department of Clinical Neurosciences, University of Oxford, Oxford, UK; 6 Department of Neurosurgery, Seoul National University Hospital, 101, Daehak-ro, Jongno-gu, Seoul 03080 South Korea

**Keywords:** LGI1, LGI1 encephalitis, Autoantibody, Human brain asymmetry, Brain lateralization

## Abstract

**Background:**

While brain asymmetry has been a fascinating issue in neuroscience, the critical mechanism remains to be elucidated. Based on some index cases with asymmetric 18F-fluoro-2-deoxy-d-glucose positron emission tomography (FDG-PET) uptake in leucine-rich glioma-inactivated 1 (LGI1)-antibody encephalitis, we hypothesized LGI1 expression could be asymmetrically distributed in the human brain.

**Methods:**

We enrolled 13 patients who were diagnosed with LGI1-antibody encephalitis between June 2012 and January 2018 at Seoul National University Hospital. Their pretreatment 18F-FDG-PET images were analyzed to find asymmetry between the left and right hemispheres. Guided by these observations, expression of LGI1 in the human hippocampus and the globus pallidus of both cerebral hemispheres was studied in nine post-mortem human brains.

**Results:**

Eleven of the 13 LGI1-antibody encephalitis patients (84.6%) showed asymmetrical FDG high uptake in the hippocampus: nine (81.8%) on the left hippocampus and two (18.2%) on the right. In the basal ganglia, seven patients (53.8%) showed asymmetry: four (57.1%) on the left and three (42.9%) on the right. The asymmetry was not evident in the laterality of faciobrachial dystonic seizures, brain MRI, and EEG. When the expression of LGI1 protein was analyzed in nine post-mortem human brains by western blotting, LGI1 expression was higher on eight left globus pallidus samples (88.89%, *P* = 0.019) and on four left hippocampal samples (44.44%, *P* = 0.652), compared to their right hemisphere samples.

**Conclusions:**

Imaging parameters from patients with LGI1-antibody encephalitis and studies of LGI1 protein expression suggest that LGI1 is asymmetrically distributed in the human brain. These observations have implications for our understanding of human brain development.

**Electronic supplementary material:**

The online version of this article (10.1186/s12974-018-1314-2) contains supplementary material, which is available to authorized users.

## Background

Brain asymmetry, or the lateralization of brain function, has been a fascinating area in neuroscience. After the first discovery of the laterality of the language center in the nineteenth century [1, 2], it has been known that the human brain has laterality of the left and right hemispheres, as the human body does. According to the studies in various species, the molecular mechanism breaking the symmetry of the body axis is based on the difference of expression of specific molecules [3]. In humans, although the recent progress in genetic studies has suggested candidate molecules [4], the critical mechanisms involved in establishing human brain asymmetry remain to be elucidated.

Leucine-rich glioma-inactivated 1 (LGI1)-antibody encephalitis is an autoimmune synaptic encephalitis resulting in memory loss, psychiatric symptoms, and faciobrachial dystonic seizure (FBDS) [5–9]. LGI1 is one of the potassium channel complex proteins that binds to ADAM22 and ADAM23 [5, 8], and the major disruptions of LGI1 in LGI1-antibody encephalitis involve the hippocampus and the motor cortex [10]. Because the LGI1 antibody disrupts the interaction between LGI1 and ADAM22 with decreasing synaptic expression of anti-α-amino-3-hydroxy-5-methyl-4-isoxazolepropionic acid (AMPA) receptor [5, 11, 12], the diseases can be unique human models for the analysis of LGI1 function. It has been reported that 18F-fluoro-2-deoxy-d-glucose positron emission tomography (FDG-PET) scan in patients with LGI1-antibody encephalitis has asymmetric uptake in the hippocampus and the basal ganglia [13]. Interestingly, experimental evidence has suggested that LGI1 is involved in brain development in mouse models [14, 15]. However, whether the classic brain asymmetry issue is linked to this brand new encephalitis has not been discussed yet. Finding the expression pattern of LGI1 might give new insights for understanding the clinical presentation of the disease and suggest a new clue for elucidating the development of human brain asymmetry.

Based on some index cases with strikingly asymmetric FDG-PET uptake in LGI1-antibody encephalitis, we analyzed the FDG-PET, MRI, EEG, and clinical semiology lateralization in the patients and the LGI1 expression asymmetry in human brains.

## Methods

### Patient enrolment and antibody determination

A prospective consecutive cohort of 13 patients with LGI1-antibody encephalitis was recruited between June 2012 and January 2018 at Seoul National University Hospital. Clinical notes, EEG data, and radiological data were reviewed. LGI1-antibodies were confirmed with the two methods, as described previously [16]. Briefly, these were serum and cerebrospinal fluid (CSF) assays with immunostaining of rat brain sections and a cell-based immunochemistry kit (Euroimmun AG, Lübeck, Germany). Antibodies against the contactin-associated protein-like 2 (CASPR2), and the N-methyl D-aspartate (NMDA) receptor, anti-α-amino-3-hydroxy-5-methyl-4-isoxazolepropionic acid 1 (AMPA1), AMPA2, and γ-aminobutyric-acid type B (GABA-B) receptors were negative, as were antibodies against Hu, Yo, Ri, Ma2, CV2, Amphiphysin, Recoverin, Sox1, and Titin antibodies. The Institutional Review Board of the Seoul National University approved the Autoimmune Encephalitis Cohort Study (2520140040). We received written informed consent from all patients who were registered in the cohort, and all methods were performed in accordance with the relevant guidelines and regulations.

### FDG-PET study analysis

All FDG-PET images were reviewed by three neurologists (Y. J, S-T.L, and K.C) and were further confirmed with a formal reading by two nuclear medicine radiologists. The images were mainly interpreted by visual inspection assisted with standardized uptake value at region of interests which was drawn manually. Prior to the injection of 18F-FDG (5.18 MBq/kg), all patients fasted for more than 6 h, regulating their blood sugar levels as to not exceed 210 mg/dL. Further details about the FDG-PET images are shown in Additional file 1: Table S1.

### Human brain samples and analysis of LGI1 expression

Frozen hippocampus and the globus pallidus were dissected from the left and right hemispheres of post-mortem human subjects. The samples were obtained from Sepulveda Research and the University of Miami, repositories of the NIH NeuroBioBank. The detailed information of the subjects is available in Additional file 1: Table S2. Western blotting was performed using a commercial anti-LGI1 antibody (rabbit polyclonal antibodies, ab30868, Abcam, Cambridge, MA, USA). The immunoreactions were visualized with enhanced chemiluminescence reagents (Pierce, Rockford, IL, USA) and were digitally scanned using a GS-700 scanner (Bio-Rad, Hercules, CA, USA). The optical density of the bands was analyzed with the ImageJ software (National Institutes of Health, Bethesda, MD, USA), normalized to the optical density of corresponding β-actin band, and expressed as a ratio between two hemispheres [relative LGI1 level = (left LGI level/β-actin level)/(right LGI level/β-actin level)]. If the relative LGI1 level was above 1.1 (10% increment, an arbitrary threshold), it was interpreted as LGI1 expression was asymmetrically increased on the left side.

### Statistical analysis

Binomial probability test was applied to the binary orders of categorical variables. The relative optical density level of LGI1 in western blotting was analyzed with the Wilcoxon matched pairs signed-rank test. STATA 14 (StataCorp LLC., Texas, USA) and GraphPad Prism, version 7.0 for Mac (GraphPad Software, CA, USA), were used for analyses.

## Results

### Patient characteristics

Of the 13 patients with LGI1-antibody encephalitis (Table 1), ten (76.9%) were male, and the median age was 64 years [IQR 61–72 years]. Twelve patients (92.3%) were right-handed. The most common symptom was memory impairment (12/13, 92.3%) followed by FBDS and psychiatric symptoms (9/13, 69.2%, respectively). Regarding the laboratory findings, five (5/13, 38.5%) patients had hyponatraemia (Na^+^ < 130 mEq/L). Among the 11 patients who had cerebrospinal fluid evaluations, one (9.1%) showed lymphocytosis (10 white blood cells/mL) and five (5/13, 45.5%) had mildly elevated protein profiles (> 45 mg/dL). No patient had active or previous tumor. Pretreatment brain T2-magnetic resonance imaging (MRI) scans were available for 12 patients, and electroencephalography (EEG) was performed on all the patients before the immune therapies.

**Table 1:** Clinical profiles of patients with 18F-FDG-PET images

Patient ID	Sex/age	Handedness	Clinical symptoms				Laboratory findings			Time from onset to FDG-PET	FDG-PET		Brain T2-MRI abnormality (high signal intensity)	EEG abnormality (slowing or epileptic discharge)
			Memory impairment	FBDS	GTCS	Psychiatric symptoms	Hyponatraemia (< 130 mEq/L)	CSF abnormality			Hippocampus	Basal ganglia		
								White blood cell count (white blood cells/mL)	Protein level (mg/dL)					
1	M/56	R	O	B				4	53	7 days	B	R	B/mild SVD	N
2	F/48	R	O	B	O	O	O	0	33	30 days	L	L	N	R
3	M/61	R	O	B	O			0	19	37 days	L	B	N	L
4	M/68	R	O			O		0	37	21 days	L	L	L/hippocampus	N
5	M/64	R		L		O		0	35	40 days	L	B	N	R
6	F/64	R	O	B				N/A	N/A	20 days	L	B	N	L
7	F/73	R	O					5	45	12 days	L	B	B/mild SVD	L
8	M/61	R	O			O		5	64	170 days	L	R	B/mild SVD	N
9	M/70	R	O	R		O		3	36	42 days	B	L	L/postcentral gyrus	B
10	M/72	R	O	O		O	O	0	68	24 days	L	B	L/hippocampus	N
11	M/74	L	O	O		O	O	10	64	37 days	R	R	N	R
12	M/74	R	O	R		O	O	N/A	N/A	2 years	L	L	N/A	L
13	M/59	R	O			O	O	0	47	8.5 month	R	B	L/hippocampus	N

*FBDS* faciobrachial dystonic seizure, *GTCS* generalized tonic-clonic seizure, *FDG-PET* 18F-fluorodeoxyglucose positron emission tomography, *MRI* magnetic resonance image, *EEG* electroencephalography, *SVD* small vessel disease, *R* right, *L* left, *b* = bilateral or no laterality, *N* normal, *O* presence, *N/A* not available data

Empty cells indicate normal or absence; In FBDS, O indicates the undecidable laterality due to the insufficient medical records (patients 10 and 11 were described as having twitching along the face and neck, but the laterality whether the FBDS was left or right was not documented)

### Asymmetry of FBDS, semiology, MRI, and EEG findings

Clinical features which informed asymmetry of the disease were analyzed. Among the nine patients who presented with FBDS, three (3/9, 33.3%) had unilateral attacks, with two of these on the right side, ipsilateral to their handedness. Three (33.3%) of the nine patients who showed other semiologies, such as seizures, had asymmetric localization of the hemispheres, but evidence of consistent lateralization was not definite (Additional file 1: Table S3). T2-MRI was lateralized in four patients (4/12, 33.3%), with signal hyperintensities consistently affecting the left hemisphere (three hippocampal and one cortical; postcentral gyrus). EEG showed asymmetric interictal waveforms in seven patients (7/13, 53.8%) without evidence of consistent lateralization (Table 1).

### FDG-PET asymmetry in LGI1-antibody encephalitis

**Fig. 1:**
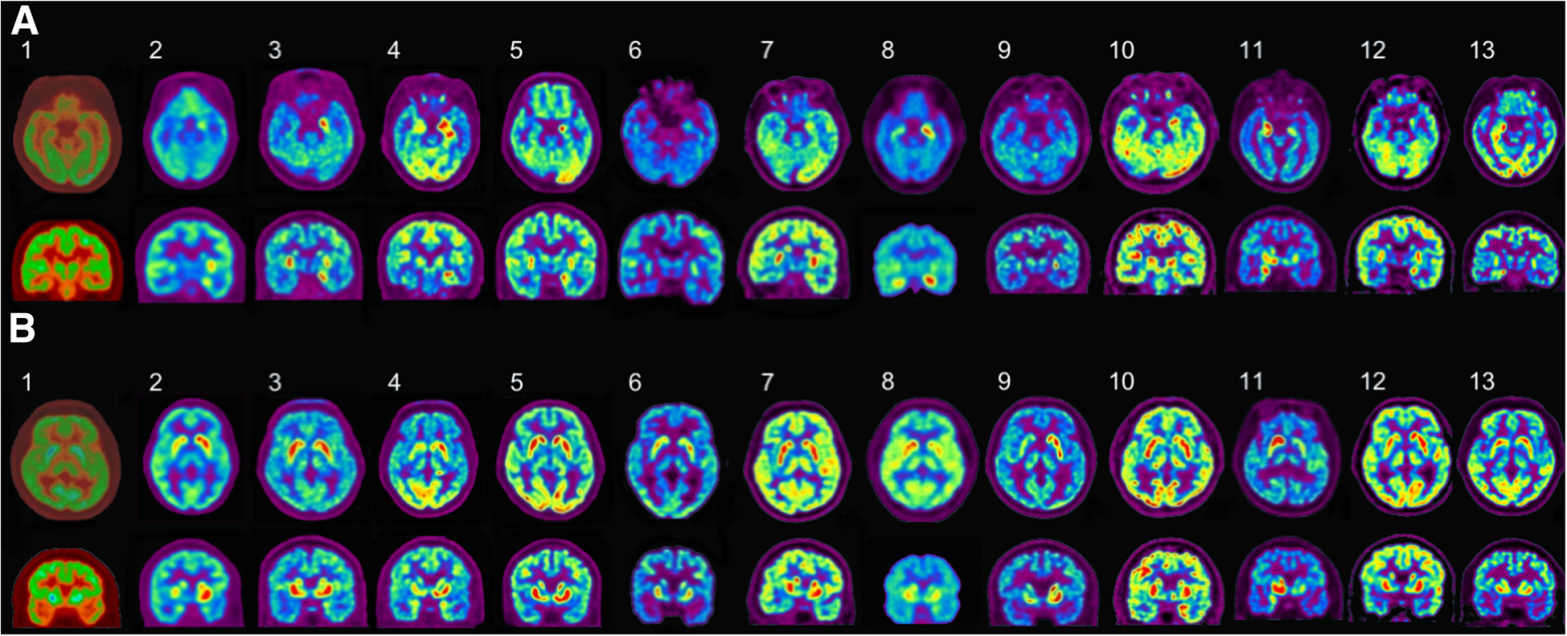
The pretreatment 18F-FDG-PET images of patients with LGI1-antibody encephalitis. **a** Axial (upper row) and coronal views (lower row) of the hippocampus: out of the 13 patients, nine (2, 3, 4, 5, 6, 7, 8, 10, and 12) had increased FDG uptake on the left side, two (11, 13) had on the right side, and two (1, 9) had no laterality. **b** Axial (upper row) and coronal views (lower row) of the basal ganglia: seven (1, 2, 4, 8, 9, 11, and 12) had asymmetric FDG uptake in the basal ganglia. Among them, four (2, 4, 9, and 12) had increase uptake on the left side, and three (1, 8, and 11) on the right side. All patients were right-handed except one (11), who was left-handed. R = right, L = left, 18F-FDG-PET = 18F-fluoro-2-deoxy-d-glucose positron emission tomography, LGI1 = leucine-rich glioma-inactivated 1

The median time duration from symptom onset to the FDG-PET image was 37 days [IQR 21–42 days]. The patients with LGI1-antibody encephalitis showed asymmetric FDG uptake that was frequently located in the hippocampus (11/13, 84.6%) and the basal ganglia (7/13, 53.9%, Table 1, Fig. 1). In the hippocampus, nine of 11 (81.8%) showed uptake of FDG on the left hippocampus and two (2/11, 18.2%) on the right hippocampus. Overall, ten (90.9%) of the 11 asymmetric hippocampus hot uptake were contralateral to patient handedness (Fig. 1; *P* = 0.035). In the basal ganglia, among the seven patients (7/13, 53.9%) who had asymmetry, five (71.4%) showed increased FDG uptake on the side contralateral to their handedness (Fig. 1), although this did not reach statistical significance (*P* = 0.647). The summarized laterality results of the subjects are available in Additional file 1: Figure S1.

### Left-dominant expression of LGI1 protein in the human brain

Given the asymmetric clinical and radiological features, we sought to determine whether the LGI1 expression is different between the left and right hemispheres. A total of 36 tissue sections from nine autopsy brains (one hippocampus and one globus pallidus tissues from each hemisphere) were obtained from two brain banks (Additional file 1: Table S2). Five of the nine brains were from right-handed donators, and four were without handedness information. In the neuropathology, seven of the nine brains were normal, one showed incidental changes consistent with aging, and one had hypoxic change without an asymmetrical feature in the cerebrum.

**Fig. 2:**
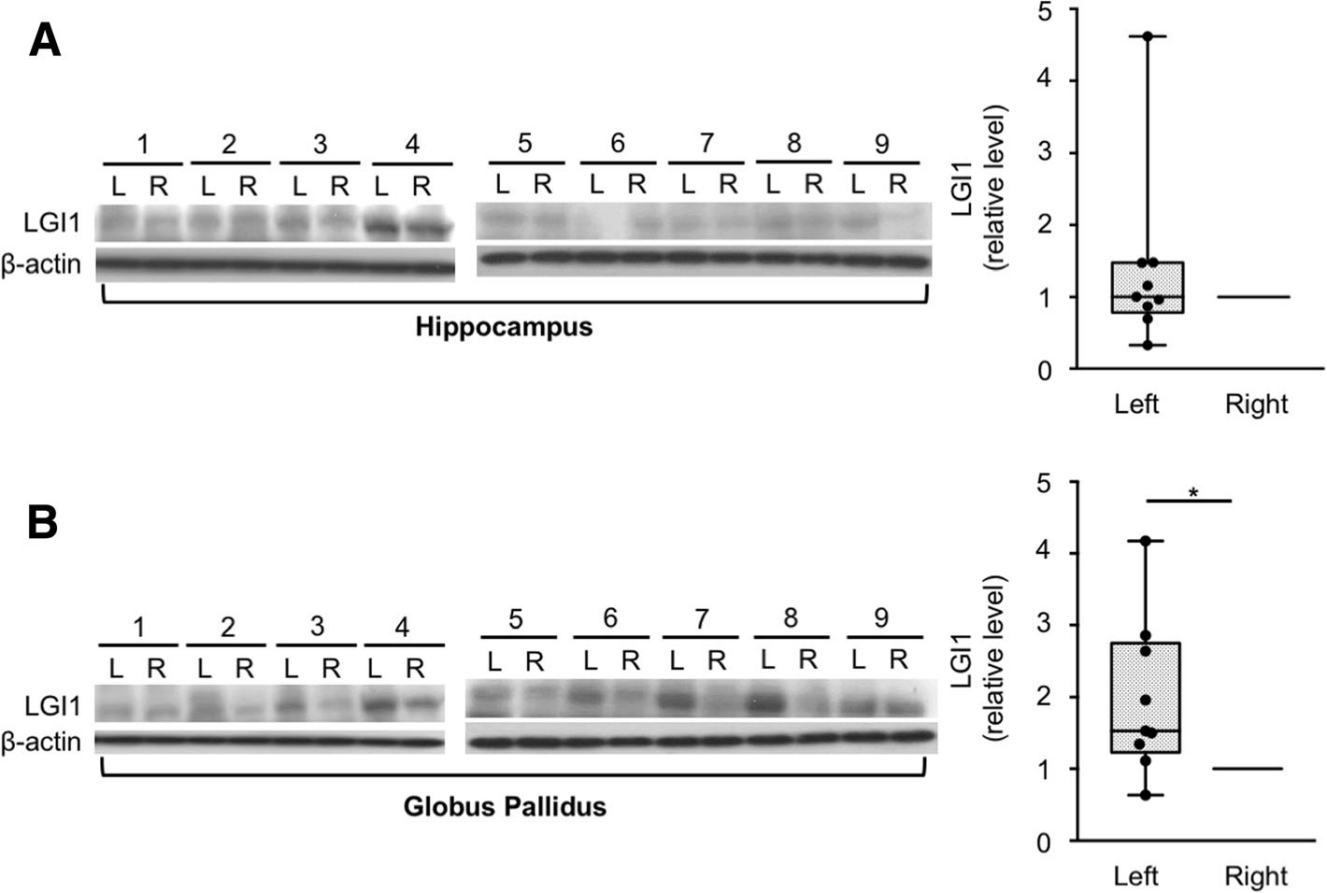
Hemispheric asymmetry of LGI1 expression in the human brain samples. **a** In the hippocampus, the western blotting and optical density analysis revealed no significant difference in LGI1 expression between both hemispheres (relative LGI1 expression of left/right = 1.39 ± 1.26, *P* = 0.653). Four samples (1, 5, 8, and 9) had higher LGI1 expression on the left side. **b** In the globus pallidus, the LGI1 expression was significantly increased on the left hemisphere (relative LGI1 expression of left/right = 1.97 ± 1.08, *P* = 0.019). All samples, except one (1), had higher LGI1 expression on the left side. The box plots show the median (line), interquartile ranges (boxes), and minimum to maximum (whiskers). *P* values were calculated using Wilcoxon matched pairs signed-rank test. *P < 0.05

In the hippocampus, the western blotting and optical density analysis revealed no significant difference in LGI1 expression between both hemispheres (relative LGI1 expression of left/right = 1.39 ± 1.26, *P* = 0.653, Fig. 2a). Four left hemisphere hippocampus showed higher expressions of LGI1 than their right hippocampus (4/9, 44.44%, in sample nos. 1, 5, 8, and 9).

However, in the globus pallidus, the LGI1 expression was significantly increased on the left hemisphere (relative LGI1 expression of left/right = 1.97 ± 1.08, *P* = 0.019, Fig. 2b). Eight left globus pallidus samples (8/9, 88.89%, in sample nos. 2, 3, 4, 5, 6, 7, 8, and 9) showed higher expressions of LGI1 compared to their right globus pallidus. This result suggested that the LGI1 expression is asymmetric (left > right) in the human brain.

## Discussion

This is the first study to reveal the asymmetry of LGI1 protein expression in the human brain; the patients with LGI1-antibody encephalitis had asymmetric FDG-PET images displaying increased FDG uptake on the contralateral hemisphere to the handedness (the dominant hemisphere), and the human brain samples expressed more LGI1 protein on the left globus pallidus (possibly dominant side).

Human LGI1 gene mutation induces autosomal dominant partial epilepsy with auditory features (ADPEAF), a rare form of familial temporal epilepsy [17]. Interestingly, according to the literature reviews (Additional file 1: Table S4), the patients with ADPEAF had a tendency to have an abnormality on the left hemisphere (probably the dominant hemisphere) in various tests, including EEG, auditory-evoked potentials, MRI, and functional MRI (fMRI) [18–23]. Nevertheless, while several previous studies analyzed the gene expression asymmetry in human brains [24, 25], the laterality of LGI1 protein expression in the human brain has not been observed thus far. Because the difference in LGI1 expression between the left and right hemispheres is specifically localized to deep structures, such as globus pallidus, as shown in our study, it would have been difficult to detect the changes in the former screening tests for asymmetry of the human brain, which were performed in the cortex.

Our results implied that LGI1 protein could be one of the determinants of human brain asymmetry. While LGI1 participate in brain development [14, 26], the exact role has not yet been determined. One of the suggestions is that LGI1 might be involved in the canonical axonal guidance pathway [27]. Because the invasion and cell migration of glioma cells are suppressed by overexpression of LGI1, it is likely that the cytoarchitecture and developmental cell migration could be asymmetric between the left and right hemispheres due to the difference in the amount of LGI1 expression. Interestingly, the patients with ADPEAF had cortical malformations at the left temporal lobe in MRI [20, 23] as shown in Additional file 1: Table S4. A further research on gene transcription in embryonic human left and right subcortical brain could support our hypothesis. Moreover, it would be valuable to observe the anatomical and functional difference of LGI1 expression between the two hemispheres in animal models, such as mice and rats.

LGI1 might be a significant factor in the determination of dominant hemisphere in humans. Currently, handedness and language lateralization are believed to have multifactorial determinants including genetic and non-genetic components [3]. With respect to genetics, leucin-rich repeat transmembrane neuronal 1 (LRRTM1) is known to be one of the key molecules associated with handedness [28]. It might not be just a coincidence that LRRTM1 and LGI1 share leucine-rich repeat domains [26]. According to our literature reviews, all the subjects with LGI1 mutation had altered language processing in fMRI analysis [19], suggesting left hemisphere abnormality. In addition, our data showed that almost all the patients with LGI1-antibody encephalitis who showed asymmetry in the FDG-PET images, except one, had increased FDG uptake in the hippocampus on the contralateral side of their handedness. Particularly, patient 11 was a left-handed person, and he had a high uptake of FDG on the right hippocampus. Moreover, the only one (patient 13), who showed a high uptake of 18F-FDG on the ipsilateral right-side hippocampus with his right handedness, also had high signal intensity on the left hippocampus in the subsequent T2-brain MRI, which was taken 8.5 months before the FDG-PET images. This patient did not receive appropriate immune therapies, and then, the follow-up MRI at the time point of FDG-PET revealed atrophy of the left hippocampus with newly developed high signal intensity on the right hippocampus (Additional file 1: Figure S2). Accordingly, it could be possible that the initial pathology of patient 13 occurred on the left hippocampus, and after the disease progressed, the FDG uptake on the left hippocampus was diminished due to the loss of the LGI1-bearing neurons, resulting in the loss of the laterality. This hypothesis needs experimental evidence.

Pretreatment 18F-FDG-PET was the uniquely sensitive investigation tool in detecting the asymmetry of LGI1 protein in the patients with LGI1-antibody encephalitis. Noticeably, the laterality was emphasized in the hippocampi of the patients, while the laterality was demonstrated in the globus palliduses in the autopsy samples. Because 18F-FDG-PET displays hypermetabolism of neurons and glial cells by the binding of the LGI1 antibodies, it does not directly show the expression of LGI1 protein. Thus, the laterality of the hippocampus in terms of FDG-PET might be due to the secondary effect of the asymmetric activation of the basal ganglia, which is connected to the hippocampus. On the premise that a case has no ethical problem, biopsy or autopsy of basal ganglia and hippocampus at bilateral hemispheres in the patient with LGI1-antibody encephalitis would be critical to verify the hypothesis. It is also our limitation that the FDG-PET images were not analyzed in a quantitative manner. Although the standardized uptake value was helpful to identify the asymmetry of FDG uptake between the left and right hemispheres, no absolute criteria for abnormalities have been established in neuroinflammation. Further quantitative analysis would be valuable for verifying a diagnostic reference of FDG-PET imaging in LGI1-antibody encephalitis.

Other clinical features including the clinical semiology, MRI, and EEG were not sensitive enough to show the asymmetry of LGI1 protein expression in the patients with LGI1-antibody encephalitis. As known to most frequently involve either side [7], the bilateral type of FBDS was the most common in our data. The bilateral FBDS might be explainable if the phenomenal threshold is low enough in basal ganglia, even though the inflammation is asymmetric. If the number of the involvement in each side for every single FBDS is counted in the individuals, it might be possible to find the asymmetry of FBDS even in those with the bilateral type of FBDS. Further prospective studies are needed to validate this hypothesis.

## Conclusion

In conclusion, our study provided the novel characteristics of LGI1 expression in the human brain by showing the asymmetry of FDG-PET images in LGI1-antibody encephalitis and the asymmetric LGI1 expression in the human brain samples. Although our study included a small number of tissue samples, has FDG-PET results only from the Asian ethnicity, and did not identify a gene-functional causal relationship, the study of the neurobiological roles of LGI1 in the developing brain is warranted, especially in terms of human brain asymmetry and handedness.

## Additional file

Additional file 1: **Table S1.** Detailed profile of the 18F-FDG-PET scanners for patients with LGI1-antibody encephalitis. **Table S2.** Detailed profile of the autopsy samples. **Table S3.** Semiology of the patients with LGI1-antibody encephalitis. **Table S4.** Overall literature review of asymmetric abnormality in patients with ADPEAF. **Figure S1.** Summary of pretreatment clinical and laboratory laterality in patients with LGI1-antibody encephalitis. **Figure S2.** T2-weighted FLAIR MRI of patient 13. (DOCX 181 kb)

## Abbreviations

ADPEAF: Autosomal dominant partial epilepsy with auditory features
AMPA: Anti-α-amino-3-hydroxy-5-methyl-4-isoxazolepropionic acid
CASPR2: Contactin-associated protein-like 2
EEG: Electroencephalography
FBDS: Faciobrachial dystonic seizure
FDG-PET: 18F-Fluoro-2-deoxy-d-glucose positron emission tomography
fMRI: Functional MRI
GABA-B: γ-Aminobutyric-acid type B
LGI1: Leucine-rich glioma-inactivated 1
LRRTM1: Leucin-rich repeat transmembrane neuronal 1
MRI: Magnetic resonance imaging
NMDA: N-methyl D-aspartate receptors
