# Supplementary material for: LGI1 expression and human brain asymmetry: insights from patients with LGI1-antibody encephalitis
Source: J Neuroinflammation. 2018 Sep 25;15:279. doi: 10.1186/s12974-018-1314-2 (PMC6156957; doi:10.1186/s12974-018-1314-2)
Supplement: Supplementary file 1 — Table S1. Detailed profile of the 18F-FDG-PET scanners for patients with LGI1-antibody encephalitis. Table S2. Detailed profile of the autopsy samples. Table S3. Semiology of the patients with LGI1-antibody encephalitis. Table S4. Overall literature review of asymmetric abnormality in patients with ADPEAF. Figure S1. Summary of pretreatment clinical and laboratory laterality in patients with LGI1-antibody encephalitis. Figure S2. T2-weighted FLAIR MRI of patient 13. (DOCX 181 kb) [file 12974_2018_1314_MOESM1_ESM.docx]

| **Table S1. Detailed profile of the 18F-FDG PET scanners for patients with LGI1-antibody encephalitis** | | |
| --- | --- | --- |
| Patient ID | 18F-FDG PET Scanner | Institution |
| 1 | Discovery STE (General Electric Medical Systems, Milwaukee, WI, USA) | Seoul National University Bundang Hospital |
| 2 | Biograph 40 (Siemens Medical Solutions USA, Knoxville, TN, USA) | Seoul National University Hospital |
| 3 | SOMATOM Definition AS (Siemens healthcare, Erlangen, Germany) | Seoul National University Hospital |
| 4 | SOMATOM Definition AS (Siemens healthcare, Erlangen, Germany) | Seoul National University Hospital |
| 5 | SOMATOM Definition AS (Siemens healthcare, Erlangen, Germany) | Seoul National University Hospital |
| 6 | SOMATOM Definition AS (Siemens healthcare, Erlangen, Germany) | Seoul National University Hospital |
| 7 | Biograph 40 (Siemens Medical Solutions USA, Knoxville, TN, USA) | Seoul National University Hospital |
| 8 | Allegro (Philips Medical Systems, Cleveland, OH, USA) | Korea University Ansan Hospital |
| 9 | Biograph 40 (Siemens Medical Solutions USA, Knoxville, TN, USA) | Seoul National University Hospital |
| 10 | Biograph 64 (Siemens Medical Solutions USA, Knoxville, TN, USA) | Seoul National University Hospital |
| 11 | Biograph 40 (Siemens Medical Solutions USA, Knoxville, TN, USA) | Seoul National University Hospital |
| 12 | SOMATOM Definition AS (Siemens healthcare, Erlangen, Germany) | Seoul National University Hospital |
| 13 | Biograph 64 (Siemens Medical Solutions USA, Knoxville, TN, USA) | Seoul National University Hospital |
| 18F-FDG PET=18F-fluorodeoxyglucose positron emission tomography; | | |

| **Table S2. Detailed profile of the autopsy samples** | | | | | | |
| --- | --- | --- | --- | --- | --- | --- |
| Sample ID | Institution | Age | Sex | Handedness | Neuropathology | Autolysis time (hours) |
| 1 | **Sepulveda Research** | 61 | M | N/A | Normal | 19.5 |
| 2 | **Sepulveda Research** | 77 | M | Right | Hypoxic change, intermediate, cerebrum | 15.5 |
| 3 | **Sepulveda Research** | 59 | M | N/A | Normal | 20.3 |
| 4 | **Sepulveda Research** | 72 | M | Right | Aging, incidental changes consistent with aging | 12.2 |
| 5 | University of Miami | 50 | M | N/A | Normal | 16.5 |
| 6 | University of Miami | 75 | M | Right | Normal | 14.2 |
| 7 | University of Miami | 71 | F | N/A | Normal | 16.4 |
| 8 | University of Miami | 79 | F | Right | Normal | 17.8 |
| 9 | University of Miami | 30 | M | Right | Normal | 20.8 |
|  | | | | | | |

| **Table S3 Semiology of the patients with LGI1-antibody encephalitis** | | |
| --- | --- | --- |
| Patient ID | Semiology | Hemisphere Localization |
| 1 | Ictal speech |  |
| 2 | Secondary GTCS, starting with left eyeball deviation | Right |
| 3 | Secondary GTCS, starting with right hand tonic posture | Left |
| 4 | Myoclonus at both legs (Right>Left) | Bilateral |
| 5 | Déjà vu, gelastic seizure |  |
| 6 |  |  |
| 7 | Right eyeball deviation, Right neck version | Left |
| 8 | Goose bump at whole body |  |
| 9 |  |  |
| 10 | Dystonic posture at both hands | Bilateral |
| 11 | Lip smacking, febrile sensation at face |  |
| 12 |  |  |
| 13 |  |  |

| **Table S4 Overall literature review of asymmetric abnormality in patients with ADPEAF** | | |
| --- | --- | --- |
|  | EEG (No./No.) | MRI (No./No.) |
| Di Bonaventura et al. (2009) | Left (3/3, 100%) |  |
| Ottman et al. (2008) |  | Left^*^ |
| Tessa et al. (2007) |  | Left^†^ |
| Brodtkorb et al. (2005) | Left (8/8, 100%)^‡^ |  |
| Pizzuti et al. (2003) | Left (2/4, 50%) vs Bilateral (1/4, 25%) vs Normal (1/4, 25%) |  |
| Kobayashi et al. (2003) |  | Left (10/22, 45.5%) vs Normal (12/22, 54.5%) |
| Left and right indicate the abnormal hemisphere. ADPEAF=autosomal dominant partial epilepsy with auditory features; EEG=electroencephalography; MRI=magnetic resonance imaging; No.= number of subjects; Empty cells indicate non-lateralized result or not available data; *functional MRI analysis during auditory description decision task in 8 patients compared with 20 healthy control subjects; †voxel-based analysis in 8 patients compared with 24 healthy control subjects; ‡Auditory evoked potentials analysis. | | |


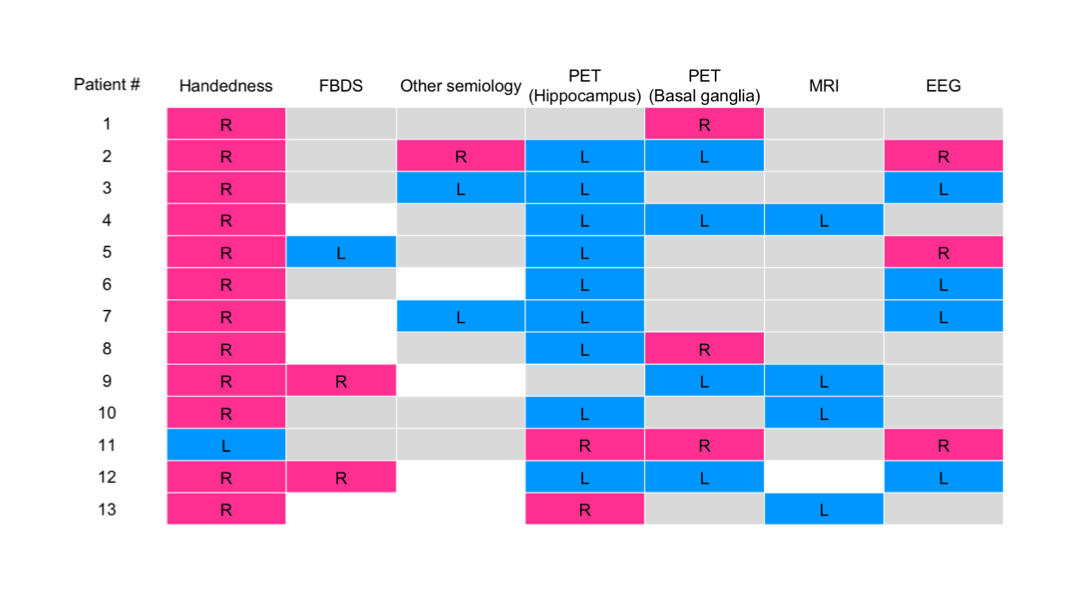


**Figure S1 Summary of pre-treatment clinical and laboratory laterality in patients with LGI1-antibody encephalitis**. Each row indicates patients with LGI1-antibody encephalitis, and each column indicates the clinical and diagnostic information of the patients. Colour indicates the laterality of each item in the column: pink=right side; blue=left side; grey=no laterality; white=no symptoms or not available. R=right; L=left; FBDS=faciobrahial seizure.


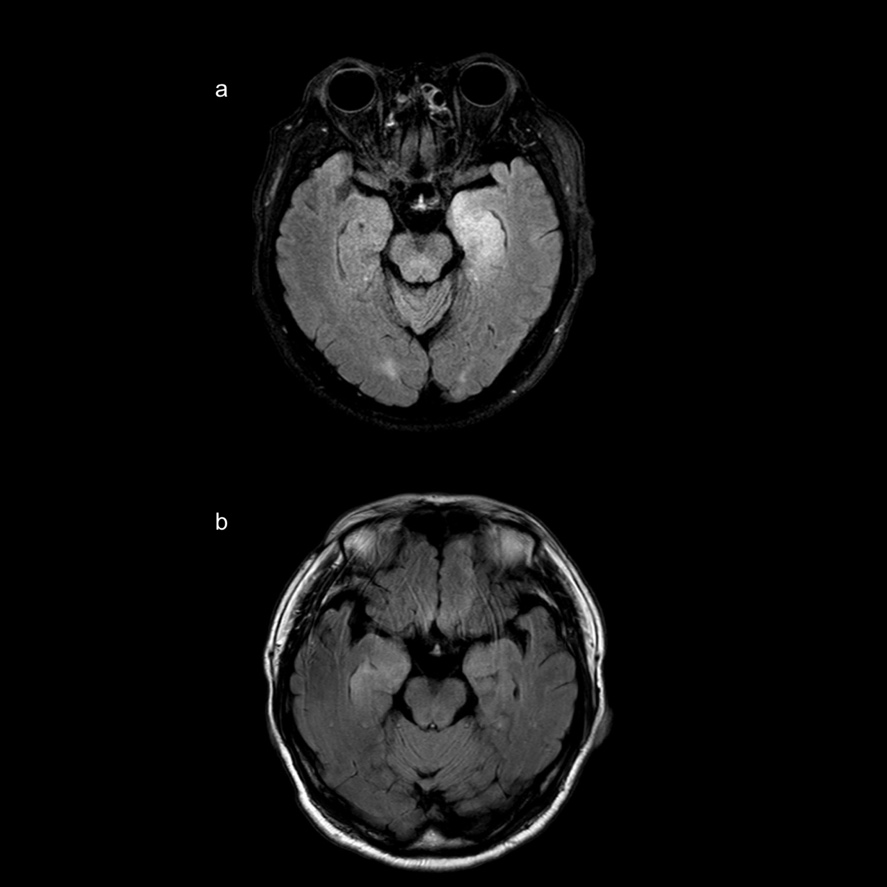


**Figure S2 T2-weighted FLAIR MRI of Patient 13.** (A) Patient 13 initially showed high signal intensity in the left hippocampus. (B) After 8.5 months without immune therapy (at the time point of FDG-PET), the follow-up images revealed atrophy of the left hippocampus and newly developed high signal intensity in the right hippocampus.
